# Comment on “Extracellular Vesicles Slow Down Aβ(1–42) Aggregation by Interfering with the Amyloid Fibril Elongation Step”

**DOI:** 10.1021/acschemneuro.4c00601

**Published:** 2024-10-09

**Authors:** Mohsin Shafiq, Andreu Matamoros-Angles, Sussane Caroline Meister, Markus Glatzel

**Affiliations:** Institute of Neuropathology, University Medical Center Hamburg-Eppendorf, Hamburg 20246, Germany

**Keywords:** Extracellular vesicles (EVs), amyloid-β (Aβ), aggregation, cellular prion protein (PrPC), Alzheimer’s disease, thioflavin-T (ThT) assay

## Abstract

Halipi et al. explored the impact of extracellular vesicles (EVs) on amyloid-β (Aβ) aggregation. They concluded that EVs reduce Aβ aggregation, as seen by shorter and thicker fibrils. While we agree with the complex role of EVs in Alzheimer’s disease, we are sceptical of the claim that EVs slow down Aβ aggregation, noting missing key references. Previous literature rather suggests that EVs (derived from neuronal cell lines) accelerate the process of Aβ fibrillation and plaque formation. Halipi et al.’s findings may be skewed due to the lack of essential neuronally expressed Aβ-binding partners, like the prion protein (PrP^C^) in their EV samples. The commentary, in the light of included original experiments and cited literature, suggests that membrane proteins like PrP^C^ are crucial to fully understand the role of EVs in Aβ aggregation, and Halipi et al.’s conclusions should be reexamined in light of these factors.

## Introduction

Halipi et al.^1^ addressed the important question of the influence of extracellular vesicles (EVs) on the kinetics of the buildup of amyloid-β (Aβ) in Alzheimer’s disease. For this, they followed a reductionist approach using EVs isolated from neuronal (SH-SY5Y neuroblastoma) and non-neuronal (HEK293-T embryonic kidney) cells incubated with recombinant Aβ(1–42) peptide and used thioflavin-T (ThT) fluorescence assays, atomic force microscopy, and cryogenic electron microscopy to monitor aggregation. The authors observed that the presence of EVs reduced Aβ(1–42) aggregation by inhibiting fibril elongation, resulting in shorter and thicker amyloid-β fibrils. While we fully agree with the conclusions of Halipi et al. that EVs play important roles in Alzheimer’s disease and that their role is complex, i.e., spreading disease pathology but also ameliorating disease by trapping neurotoxic Aβ species, we do not agree with the statement that EVs slow down Aβ(1–42) aggregation. Furthermore, we think that key references in the field were omitted, not describing the full picture of what is known about the role of EVs in Aβ aggregation.

Besides the studies referenced by Halipi et al., numerous publications have focused on the interplay of EVs and amyloid-β. One of the first papers on the subject,^2^ which was cited by Halipi et al., already suggested that EVs precipitate Aβ and thus lead to or accelerate Aβ plaque formation. Follow-up papers showed that inhibition of EV biogenesis reduces Aβ plaque load^3^ and incubation of EVs with Aβ increases its fibrillation.^4^ In fact, EV-bound Aβ in blood correlates with Aβ plaque load in AD patients.^5^

Also, Halipi et al. state that certain types of lipids, including phosphatidylserine and monosialoganglioside, have been reported to exhibit inhibitory effects on Aβ aggregation. For this assertion, the authors cite an article by Sanguanini et al., 2020.^6^ However, Sanguanini et al. also suggested that once a mixture of phospholipids is present, the inhibitory effect toward Aβ aggregation disappears. Given that EVs, particularly those derived from the brain, have a complex lipid composition, containing varying amounts of glycerophospholipids, glycerophosphocholine, and glycerophosphoethanolamine,^7,8^ the balanced lipid content found in EVs may not significantly modulate Aβ aggregation.

## Results and Discussion

**Figure 1:**
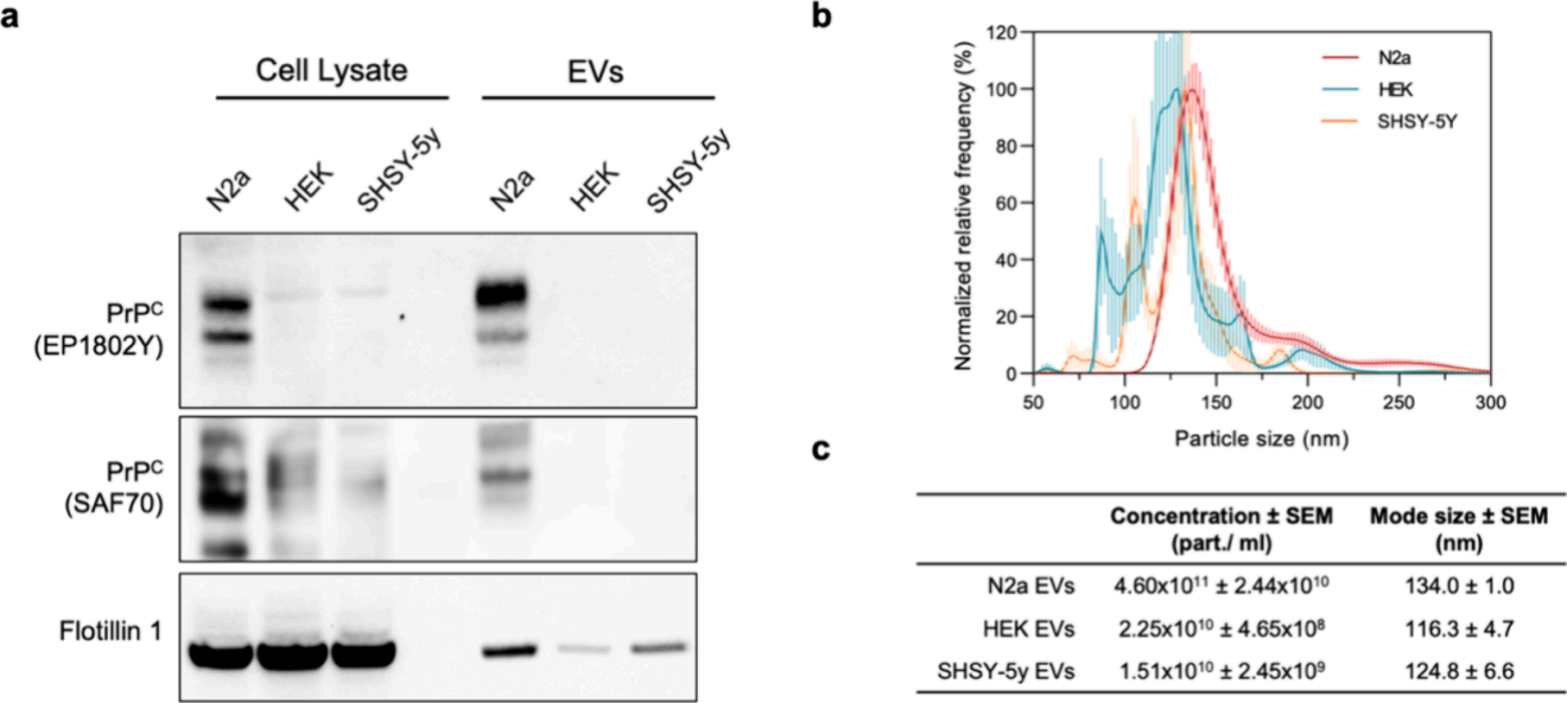
Characterization of PrP^C^ expression in HEK293, SHSY-5Y, and Neuro-2a cell lysates and derived EVs. (a) Western blot analysis of lysates and EVs from HEK293, SHSY-5Y, and Neuro-2a cells showing a signicantly low PrP^C^ expression in the lysates from HEK293 and SHSY-5Y compared to that of Neuro-2a cells, and the absence of detectable amounts of PrP^C^ in HEK and SHSY-5Y derived EVs compared to the EVs isolated from Neuro-2a cells. The PrP^C^ expression is validated with two different anti-prion antibodies, EP1802Y and SAF70. The expression of Flotillin-1 confirms the enrichment of EVs in the samples. The cell lysates are normalized by protein concentration and the EV samples by the number of EVs measured by nanoparticle tracking analysis (NTA). (b) Percentage size distribution of EVs derived from Neuro-2a, HEK293, and SHSY-5Y by NTA. (c) Mean concentration (particles/mL) and mode size (nm) of the EVs isolated from HEK293-T, SHSY-5Y, and Neuro2a cells were measured by NTA.

How can the discrepancy between the data of Halipi et al. and the above-mentioned facts be reconciled? One explanation may be that the EVs isolated by Halipi et al. are missing essential partners required for Aβ binding and subsequent fibrillation. Known binding partners leading to Aβ binding and subsequent fibrillation include the cellular prion protein (PrP^C^)^9−11^ and certain glycosphingolipids.^12^ We assessed levels of PrP^C^ in the lysates of SH-SY5Y and HEK293-T cells and compared them to PrP^C^ levels of a neuronal cell line, the Neuro-2a cells. In both SH-SY5Y and HEK293-T cells, PrP^C^ levels were significantly low compared to the levels in the Neuro-2a cells (Figure 1a and Falker et al., 2016^10^). Consequently, we isolated EVs from the three cell lines using similar methods as Halipi et al. and assessed levels of PrP^C^ in EV preparations. As expected, the PrP^C^ levels in the EVs isolated from SH-SY5Y and HEK293-T were below the detection limit of the assay, in contrast to the Neuro-2a EVs (Figure 1b and Falker et al. 2016^10^). This lack of PrP^C^ expression on SH-SY5Y and HEK293-T EVs may explain the discrepancies observed by Halipi et al. in regard to the modulatory behavior of EVs in Aβ aggregation.

In summary, in our opinion, the conclusions drawn by the authors, particularly the broad assertion that EVs slow down Aβ aggregation, need a critical reevaluation not only because of the novel data presented in this commentary but also given the omission of important data and contextualization on the shown data. Crucial factors, such as membrane receptor proteins like PrP^C^, play a significant role in the Aβ aggregation, and they should be under consideration when analyzing this complex phenomenon.

## Methods

### Extracellular Vesicles Isolation

Gibco DMEM media supplemented with 10% exosome-depleted FBS (EXO-FBS-50A-1, System Bioscience) were conditioned with HEK293, SH-SY5Y, and Neuro-2a cells for 48 h. Media were centrifuged at 200*g* for 5 min, 7000*g* for 20 min, passed through a 0.22 μm filter, and finally centrifuged at 100,000*g* for 70 min in an Optima L-100 XP ultracentrifuge using a SW40Ti rotor (Beckman Coulter, Brea, CA, USA). The EV pellets were resuspended in phosphate buffered saline (PBS, Thermo Fisher Scientific) containing EDTA-free protease inhibitor cocktail (complete EDTA-free tablet, Roche) and used for further experiments (including nanoparticle tracking analysis and Western blotting).

### Nanoparticle Tracking Analysis

EVs were quantified and characterized using a NanoSight LM14 (Malvern). Samples were diluted with PBS 1/250 or 1/500 for the measurements. Five videos of 30 s were recorded and processed with the NanoSight NTA 3.0 software to determine the size distribution and particle concentration.

### Western Blotting

Cells were washed with PBS and lysed in an appropriate amount of RIPA buffer (50 mM Tris base, 150 mM NaCl, 1% NP40, 0.5% Na-deoxycholate, and 0.1% SDS at pH = 8) containing protease inhibitor cocktail, incubated on ice for 10 min, centrifuged at 15,000*g* for 10 min, and the protein concentrations of supernatants (=lysates) were determined by Bradford assay (Bio-Rad Laboratories). The desired amount of total protein was mixed with 4× sample buffer (240 mM Tris base, 8% SDS, 40% glycerol, and 0.2% bromophenol blue at pH = 6.8, containing 5% β-mercaptoethanol), incubated at 95 °C for 5 min. Similarly, normalized volume of EV samples were also mixed with 4× sample buffer (240 mM Tris base, 8% SDS, 40% glycerol, and 0.2% bromophenol blue at pH = 6.8, containing 5% β-mercaptoethanol), incubated at 95 °C for 5 min. After the incubation at 95 °C, samples were loaded in precasted 4–12% Bis-Tris protein gels (Invitrogen). Proteins were transferred to nitrocellulose membranes (Bio-Rad) and detected using mouse monoclonal antibodies against PrP^C^, clone EP1802Y (#ab52604, Abcam) and clone SAF70 (#189770, Cayman Chemical), and against Flotillin 1 (#610820, BD Biosciences). Corresponding HRP-conjugated secondary antibodies (anti-rabbit, #W4011, and anti-mouse #W4021, both from Promega) were incubated (1:4000) for 1 h at RT in 5% nonfat dry milk (in TBS-T buffer). Detection was performed with Pierce ECL Pico or Femto substrate (Thermo Fisher Scientific) using the Chemidoc XRS+ imaging system (BioRad).
